# Spatial regression analysis of MR diffusion reveals subject-specific white matter changes associated with repetitive head impacts in contact sports

**DOI:** 10.1038/s41598-020-70604-y

**Published:** 2020-08-12

**Authors:** Patrick D. Asselin, Yu Gu, Kian Merchant-Borna, Beau Abar, David W. Wright, Xing Qiu, Jeff J. Bazarian

**Affiliations:** 1 Department of Pediatrics, Boston Children’s Hospital, 300 Longwood Avenue, Boston, MA 02115 USA; 2 Department of Biostatistics and Computational Biology, University of Rochester, 265 Crittenden Blvd, CU 420630, Rochester, NY 14642-0630 USA; 3 Department of Emergency Medicine, School of Medicine and Dentistry, University of Rochester, 265 Crittenden Blvd, Box 655C, Rochester, NY 14642 USA; 4 Department of Emergency Medicine, Emory University, 49 Jesse Hill Jr. Drive, Atlanta, GA 30303 USA

**Keywords:** Computational models, Data processing, Outcomes research, White matter injury

## Abstract

Repetitive head impacts (RHI) are a growing concern due to their possible neurocognitive effects, with research showing a season of RHI produce white matter (WM) changes seen on neuroimaging. We conducted a secondary analysis of diffusion tensor imaging (DTI) data for 28 contact athletes to compare WM changes. We collected pre-season and post-season DTI scans for each subject, approximately 3 months apart. We collected helmet data for the athletes, which we correlated with DTI data. We adapted the SPatial REgression Analysis of DTI (SPREAD) algorithm to conduct subject-specific longitudinal DTI analysis, and developed global inferential tools using functional norms and a novel robust *p* value combination test. At the individual level, most detected injured regions (93.3%) were associated with decreased FA values. Using meta-analysis techniques to combine injured regions across subjects, we found the combined injured region at the group level occupied the entire WM skeleton, suggesting the WM damage location is subject-specific. Several subject-specific functional summaries of SPREAD-detected WM change, e.g., the ${L}^{\infty }$ norm, significantly correlated with helmet impact measures, e.g. cumulative unweighted rotational acceleration (adjusted *p* = 0.0049), time between hits rotational acceleration (adjusted *p* value 0.0101), and time until DTI rotational acceleration (adjusted *p* = 0.0084), suggesting RHIs lead to WM changes.

## Introduction

Sport-related concussions and repetitive head impacts (RHI) incurred during sports have emerged as important foci of brain injury research with the increased awareness and improved understanding of these injuries’ effects on neurocognitive function. RHI are broadly defined as any direct or indirect hit that exerts a force on the brain that may or may not cause a clinically diagnosed concussion^1^. RHI occur frequently during contact and collision sports at all levels of play including youth, collegiate, and professional competitions. Both animal and human studies have shown acute changes in brain structure and function after RHI that are indicative of axonal injury^2–10^. This understanding of the acute impact on the brain has led to questions about RHI’s potential long-term neurocognitive effects.

The direct connection between RHIs, acute brain injury, and their possible long-term effects on brain structure and neurocognitive function are difficult to confirm with retrospective research. Montenigro and colleagues showed a retrospective survey metric for head impact exposure was strongly associated with behavioral and cognitive dysfunction later in life^1^. In addition, post-mortem studies of chronic traumatic encephalopathy (CTE) have described RHI as a necessary but not sufficient risk factor, which suggests RHI lead to biologic dysregulation and clinical symptoms^11^. Despite this retrospective evidence, RHI causing CTE and other neurodegenerative diseases is considered controversial because of the lack of prospective evidence linking RHI to acute and sub-acute white matter (WM) damage^9,12^. The ability to elucidate a link between WM damage and RHI has been greatly improved with diffusion tensor imaging (DTI).

The use of DTI has been critical in identifying and quantifying the in vivo brain changes from RHI. DTI measures WM structural changes by quantifying the direction and magnitude of water diffusion in the brain^13^. Studies using DTI have shown structural changes in an athlete’s brain after a single season of RHI without frank concussion^4,14–16^. These quantifiable WM changes after a season of football without a frank concussion initially suggested a relationship with RHI. However, the studies investigating the location of injury have shown little consistency in the locations of significantly changed WM^4,17–19^. This lack of consistency cast doubt on the acute as well as the long-term effects of RHI on neurocognitive function and disease.

The methods used in these studies to compare DTI changes, region of interest analysis, and wild bootstrapping, are possible causes for the differences in regions identified in the various studies^4,17–19^. These methods are designed for group-level analyses, which work best when there is a strong spatial pattern of injury; however, the injury from RHI and sport-related concussion is thought to be subject-specific and heterogeneous^20^. The analytical shortcomings of these methods have been identified by others^21,22^. The above methods are unable to account for the variable baseline and disease progression between subjects, and they neglect the intrinsic spatial relationships in the imaging data^21,22^. The novel analysis method of SPatial REgression Analysis of DTI (SPREAD) was proposed for the longitudinal comparison of DTI within a single individual^21^. This method improves the statistical power of group-based statistical analyses by performing subject-specific DTI analysis, which accounts for the variable baseline DTI parameters and variable disease progression among individuals^21^. In addition, SPREAD accounts for the spatial correlation among the voxels, which is the concept that neighboring voxels share similar imaging or function characteristics, and accounting for this greatly improves the statistical power of DTI analysis^21^. Another advantage is SPREAD’s use of non-parametric permutation testing, which avoids some of the limitations of parametric testing and can potentially provide a more powerful and appropriate technique for analyzing subject-specific DTI changes in the setting of RHI.

This study investigated the extent to which SPREAD analysis can identify WM changes unique to contact athletes experiencing RHI; that is, we sought to identify the anatomic locations of these WM changes for each athlete, and study the overall pattern of these changes by a novel robust *p* value combination test. Secondly, we sought to determine the relationship between these WM changes to subject-specific head impact exposure over one season.

## Methods

### Participants

From 2011 to 2013, we enrolled 29 athletes, which were part of our initial data set for secondary analysis. These subjects were male varsity football players recruited during the 2013 football season by the University of Rochester, which participates in NCAA Division III. A single non-athlete subject, described elsewhere, was utilized in our simulation studies to identify optimal tuning parameters of our imaging comparison method, as described below^21,22^. The athletes were selected to include a variety of positions to capture the spectrum of head impact exposure in frequency and intensity. Subjects were excluded in the parent study if they were < 18 years old or sustained a concussion within 2 weeks of study enrollment, which was determined by a validated self-report questionnaire^25^. Data from one subject was excluded because of poor DTI image quality, leaving 28 athlete subjects for analysis, and one collegiate non-athlete subject for parameter optimization. Prior to participation in this study, all subjects provided written informed consent. The Research Subjects Review Board at the University of Rochester approved this study and the consent process. All experiments were performed in accordance with relevant guidelines and regulations.

### MR imaging acquisition and processing

Diffusion MR imaging, also known as DTI, was performed on all subjects before the start of the football season in August (pre-season) and within 1 week of the season’s end in November (post-season). All subjects had two DTI scans approximately three months apart. Imaging was acquired using a single 3 T Siemens Trio scanner (Siemens Healthcare, Erlangen, Germany) using Numaris 4 software version 17B. The matrix head coil had 32 channels, and the scanning parameters were: total acquisition time of 11 min, spin-echo echo planar imaging, bandwidth of 1502 Hz/Px, parallel acceleration technique of generalized autocalibrating partially parallel acquisition, TR/TE = 9100 ms/89 ms, voxel size 2 × 2 × 2 mm, 69 diffusion directions with b = 1200 s/mm^2^ and 10 averages of b = 0. The TR was chosen to minimize T1 weighting influences on the diffusion images and maximize the signal to noise ratio. TR times of similar duration have been used in similar studies that assess WM damage in individuals with head trauma^26^. FSL-5.0.9 was used for all preprocessing of the diffusion data (FSL; www.fmrib.ox.ac.uk). Fugue and eddy packets in FSL were used to correct for magnetic susceptibility distortions, motion, eddy currents, and brain extraction^27^. DTIFIT, an FSL packet, was used to create global maps of fractional anisotropy (FA). For time-point comparisons, the FA map for each subject’s post-season FA map was non-linearly registered to their pre-season FA map using the FSL function, FNIRT^28^. The R package “oro.nifti” was used to load NIfTI image data^29^. The registered images were noted to have ringing artifacts due to the sharp transitions near edges, which would detrimentally affect later image processing^30^. We developed a simple and computationally efficient method, known as “MountDoom” in our software package, to remove these artifacts before the subsequent analyses (Supplementary Fig. 4). We first determined the foreground (true brain signals) and the background using DTIFIT, and then removed all voxels in the foreground with Euclidean distance to the background less than or equal to a pre-specified threshold, which was set to 4 mm, and represents two voxels apart in six major directions and a 3D neighborhood with 32 voxels. After this step, we cropped the images to the individual’s smallest three-dimensional rectangle, which contained all the voxels with nonzero FA values to reduce the dimensionality and computational cost. We utilized SPREAD for image analysis.

### SPREAD analysis of DTI

**Figure 1:**
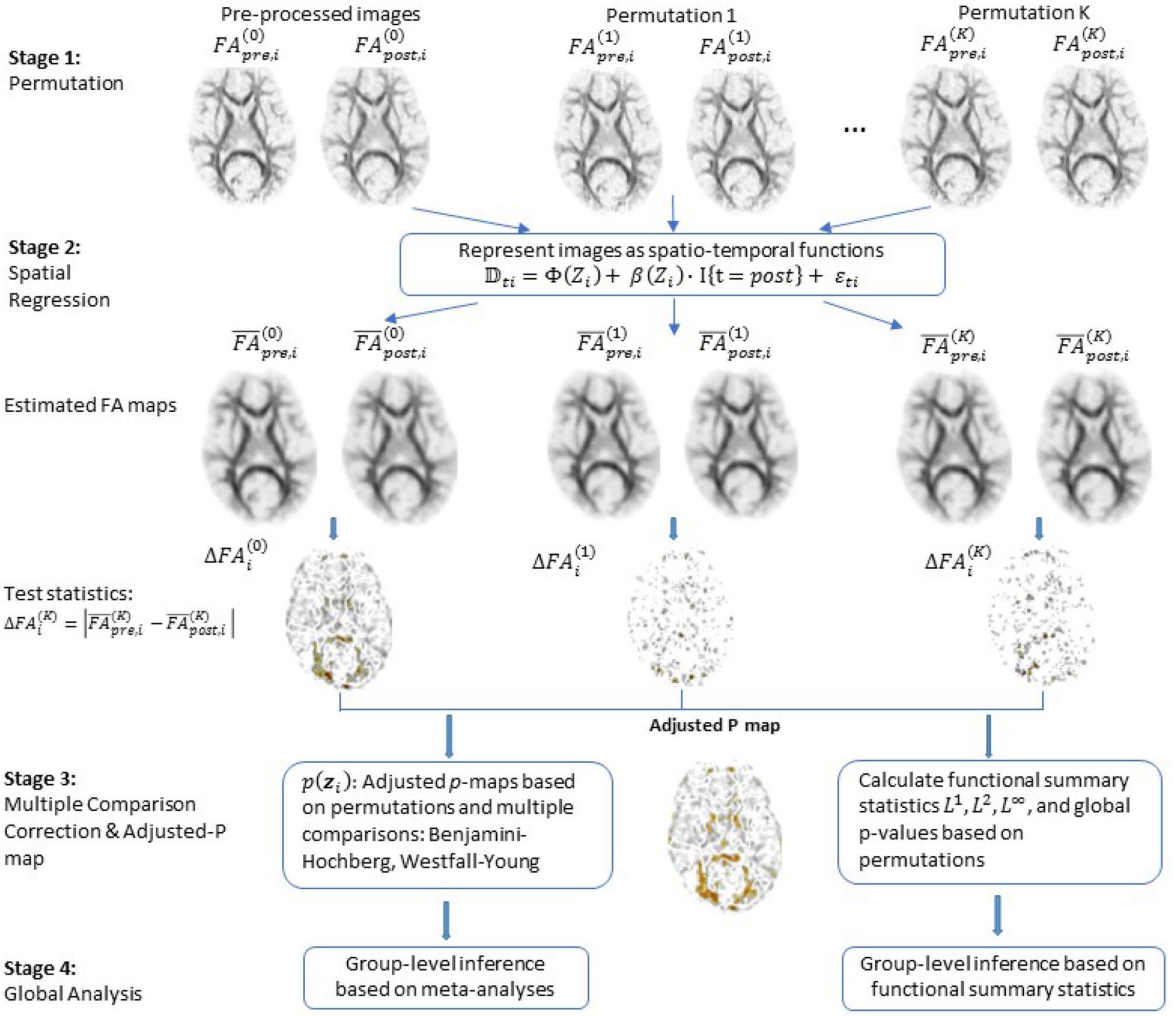
An overview of the enhanced SPREAD algorithm used in the current study**.** It depicts each stage used by the SPREAD algorithm for image analysis.

The SPREAD model used in the current study is an adaptation of the original model developed by Zhu and colleagues^21^ (Fig. 1).

**Figure 2:**
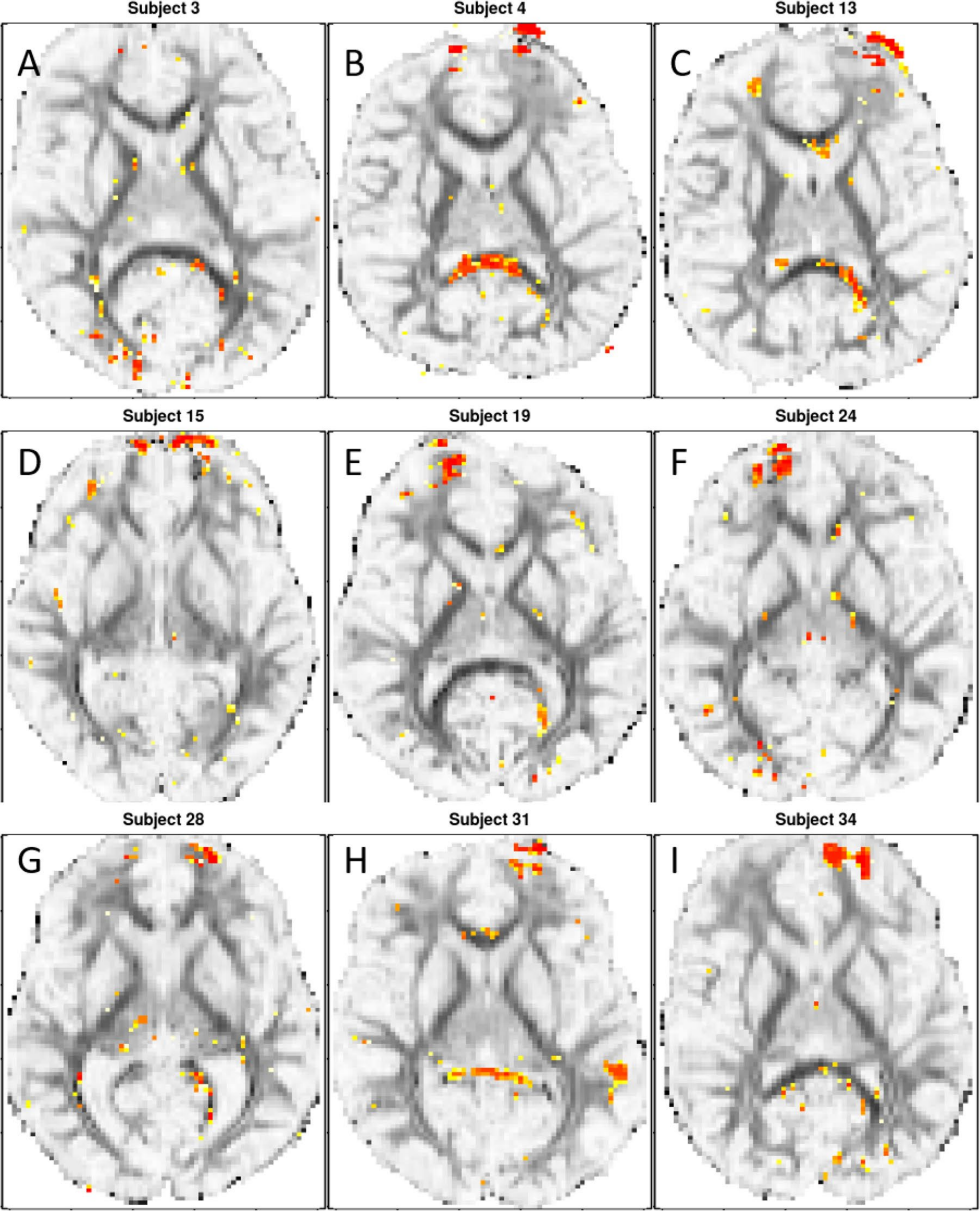
Illustrative examples of subjects’ individual *p* value map at registered slice 32. (**A**) Subject 3′s highlighted voxels at registered slice 32. (**B**) Subject 4′s highlighted voxels at registered slice 32. (**C**) Subject 13′s highlighted voxels at registered slice 32. (**D**) Subject 15′s highlighted voxels at registered slice 32. (**E**) Subject 19′s highlighted voxels at registered slice 32. (**F**) Subject 24′s highlighted voxels at registered slice 32. (**G**) Subject 28′s highlighted voxels at registered slice 32. (**H**) Subject 31′s highlighted voxels at registered slice 32. (**I**) Subject 34′s highlighted voxels at registered slice 32. The yellow/red highlighted regions of the brain are voxels with associated raw *p* values of < 0.002 when comparing the pre-season to post-season scans. The red voxels are associated with a lower raw *p* value than the yellow ones.

The original model has the capability to incorporate multiple scans collected from multiple subjects at several time points. In the original model, the voxel-wise summary statistic was calculated as the temporal standard deviation of averaged FA or mean diffusivity values for each subject, and each subject’s value was summed to get the final summary for one voxel. In our study, only the pre- and post-season scans are available for each subject and we decided to conduct subject-specific SPREAD analysis due to the high-level of heterogeneity among subjects (Fig. 2).

In this special case, the temporal standard deviation of FA values for a voxel is monotonically equivalent to the absolute value of the difference between the pre- and post-scans at this voxel, and we do not average this summary statistic over all subjects (Supplementary Methods). Using this simplification, we streamlined the code, which is mathematically equivalent to the original model. First, we randomly permuted the time labels of each voxel to create permutation samples, represented by k in our spatial regression model. We then fitted FA values, which were calculated by applying spatial regression based on multivariate Nadaraya–Watson kernel to each permutation sample. The test statistics were computed as the voxel-wise absolute difference between the fitted FA values of the pre- and post-scans for each permutation sample. Next, the permutation *p* values were computed by comparing the original, before permutation, to the absolute differences of FA values from their permuted counterparts. With the large number of hypotheses tested, we used Benjamini–Hochberg (BH) procedure to control the false discovery rate, and Westfall–Young (WY) procedure to control the family-wise error rate^30–32^. The results are adjusted *p* value maps for each subject. In addition, we calculated functional summary statistics based on L^p^ norms (see “Functional norms” in Supplementary Methods), and obtained global *p* values based on k = 1000 permutations for each subject. These global summary statistics and *p* values were used in group-level analysis. We performed group-level inferences based on associating the global functional summary statistics with clinical outcomes and combining voxel wise *p* value maps by a novel robust meta-analysis method. The SPREAD method is illustrated in Fig. 1.

### SPREAD parameter optimization

SPREAD was originally developed for detecting small WM lesions, typically under 5 voxels in size, seen with multiple sclerosis^21^. Because the size of the “abnormal” region after RHI is not known, the parameters used in the original SPREAD procedure might not be appropriate. We designed a simulation study to select the optimal tuning parameters of the SPREAD method for analyzing WM changes occurring in the setting of RHI. Specifically, we studied the performance of SPREAD with the following parameters in a series of simulations involving a pre-post image pair from a single collegiate non-athlete subject: (1) we superimposed artificial “lesions” to different regions of the post-season scan with three sizes: radius of 5 voxels (small), radius of 12 voxels (medium), and radius of 48 voxels (large); (2) various choices of bandwidth, ranging from three to 25 voxels, were used in the Gaussian kernel function; (3) BH and WY were used to obtain the adjusted *p* value maps; and (4) seven different *p* value thresholds were used to define statistically significant voxels based on the adjusted *p* value maps (1e^−4^, 5e^−4^, 1e^−3^, 5^e−3^, 0.01, 0.05, and 0.1) (Supplementary Fig. 1). We assessed true positive and false positive rates to find the optimal combination of bandwidth, multiple comparison method, and *p* value threshold for each lesion size.

### SPREAD outputs

From the SPREAD algorithm, we summarized the following outputs: number of significantly changed voxels (NSV), total difference of significant voxels (TDSV), absolute difference of significant voxels (ADSV), and the L^p^ norms of the fitted FA map. TDSV was calculated by taking the sum of the differences for significantly changed voxels between pre-season and post-season scans for a single subject. ADSV was calculated by taking the sum of the absolute difference for significantly changed voxels between pre-season and post-season scans for a single subject. L^1^ is the absolute difference between pre and post-season scans of the fitted FA values averaged over all voxels. The L^2^ norm is the square root of the average of squared difference of the fitted FA values. The L^∞^ norm is the maximum of absolute difference of the fitted FA values. TDSV, ADSV, L^1^, L^2^, L^∞^ have the units of FA, which is a unitless scalar between 0 and 1.

In addition, we conducted a meta-analysis based on a novel robust *p* value combination test based on beta-distributions. The objective of this analysis is to utilize *p* value maps, which is an image that marks voxels showing significant change from the pre and post-season scan comparison. We combine the *p* value maps of all 28 subjects into a single group-level *p* value map, and then select significant regions based on this single map (Supplementary Methods).

### Head impact exposure

Head impact exposures were estimated using the Head Impact Telemetry system (HITs) (Simbex, Lebanon, NH), which has accelerometers embedded in Riddell Revolution IQ Helmets (Riddell Corporation; Elyria, OH). HITs provides five helmet-based impact measures (HIM) for each impact > 10 g’s of linear acceleration: linear acceleration (LA), rotational acceleration (RA), Head Injury Criterion 15 (HIC 15), Gadd Severity Index (GSI), and Helmet Impact Technology severity profiles (HITsp), as well as time of impact and impact location. The 10 g threshold was chosen as it is the lowest threshold to be set by the helmets, and we were looking to record all hits that may lead to white matter damage and therefore went for the high sensitivity approach.

An equipment manager made sure all helmets were fitted to the player and connected to the computer before every contact session. A sideline computer monitored by a research coordinator at every helmeted session collected the data wirelessly. In addition, the research coordinator monitored all equipment during play, and performed daily data scrubbing to remove any recordings that occurred while the helmet was not on the subject’s head.

Accelerometer output for an entire season was summarized with six different helmet impact metrics : mean HIMs, peak HIMs, cumulative unweighted (CUW) HIMs, and three cumulative time-weighted metrics that have been previously described in detail^33^: (1) time between hits (TBH) weights HIMs for the current hit based on the magnitudes of the previous hits and the time between all previous hits and the current hit; (2) time until assessment (TUA) weights HIMs for the current hit based on the number of days between the current hit and the post-season DTI scan; (3) a combination of the TBH and TUA algorithms referred to as TBH + TUA.

Subjects undergoing RHI were monitored for concussion using methods described elsewhere in detail^33^. In brief, certified athletic trainers were present at all practices and games monitoring the athletes for concussion. Concussion was defined by the Sport Concussion Assessment Tool 2, which requires at least one of the following: symptoms (e.g. nausea), physical signs (e.g. loss of consciousness), impaired brain function (e.g. impaired memory), or abnormal behavior^34^.

### Clinical assessment of concussion-related cognitive impairment

We used the Immediate Post-Concussion and Cognitive Testing (ImPACT) (ImPACT Applications, inc) and the balance error scoring system (BESS) tests as our clinical measurements at pre-season and post-season evaluation for all 28 subjects^35,36^. The ImPACT test is a computerized test that looks at several different measurements of memory, reaction time and visual speed; from the ImPACT test, we measured verbal memory score, visual memory score, visual motor speed, and reaction time^35^. The BESS test is a balance test for which the number of errors occurring during testing are cumulated into a final score, which is what we for our subjects^36^.

### Statistical analysis

Demographic variables were reported in Table 1. For continuous variables, medians, and inter-quartile ranges (IQR) were reported; for categorical variables, sample frequencies and percentages were reported. We used a novel robust *p* value combination test to assess the anatomic location for WM changes between the subjects. The primary statistical analysis is to correlate SPREAD outputs with helmet impact metrics. Considering the uncertainty of normality of the six helmet impact metrics, Spearman's nonparametric correlation test was employed with the Benjamini–Hochberg procedure to control for multiple testing. Paired t-test was used in an auxiliary analysis to compare clinically defined, concussion-related cognitive test scores before and after the football season. We defined statistically significant findings for all testing to be those with (adjusted) *p* value < 0.05, and all tests were two-tailed. All statistical analyses were performed in R 3.3.0^37^.

**Table 1:** Subject characteristics.

	Contact athletes (n = 28)
Age (median years, IQR)	19.8, 2.1
BMI (median, IQR)	27.6, 2.1
Race, n (%)	
White	21 (75)
Black	5 (17.9)
Other	2 (7.1)
Handedness, n (%)	
Right	22 (78.6)
Left	6 (21.4)

For continuous variables (Age and BMI), medians and inter-quartile ranges (IQR) were reported; for categorical variables, sample frequency and percentages were reported.

## Results

### Demographics

The characteristics of the 28 subjects recruited in our study are summarized in Table 1. Among them, three subjects sustained a mid-season concussion (Supplementary Table 1).

### SPREAD parameter optimization

Using our simulation study to optimize the bandwidth parameter for RHI, we found the optimal bandwidth values to be five for small signal (radius of 5 voxels), 10 for medium signal (radius of 12 voxels), and 19 for large signal (radius of 48 voxels) (Table 2). We discovered that the optimal bandwidth value appears to be positively related to the signal size, i.e. the smaller the signal is, the smaller the optimal bandwidth is, which can help determine optimal bandwidth values based on lesion size created by the disease process. We also noticed that using very large bandwidth values for large signal tends to result in high levels of false positives (Supplementary Fig. 1). Based on these considerations, bandwidths of 3, 5, 10, and 15 were used in the SPREAD algorithm when analyzing the current dataset.

**Table 2:** Results of simulation study to determine the optimal bandwidth based on the signal size.

Signal Size	MTPs	Bandwidth	pValue.thresh	True positive	TPRate	False positive	FPRate
Small	BH	5	0.034	101	0.902	35	0.000
Small	WY	5	0.050	101	0.902	35	0.000
Medium	BH	10	3 × 10^–4^	1411	0.953	235	0.001
Medium	WY	10	1 × 10^–3^	1442	0.974	411	0.001
Large	BH	15	0.001	13,219	0.900	65,291	0.173
Large	WY	15	0.279	13,234	0.901	65,762	0.174
Large	BH	19	7.78 × 10^–7^	13,419	0.914	45,718	0.121
Large	WY	19	1 × 10^–5^	13,584	0.925	51,627	0.137

MTP, multiple comparison procedure; pValue.thresh, *p* value considered significant change for a voxel; True Positive, number of true positives detected; TPRate, true positive rate; False Positive, the number of false positives detected; and FPRate, the false positive rate.

### Anatomic distribution of WM changes detected using SPREAD

**Figure 3:**
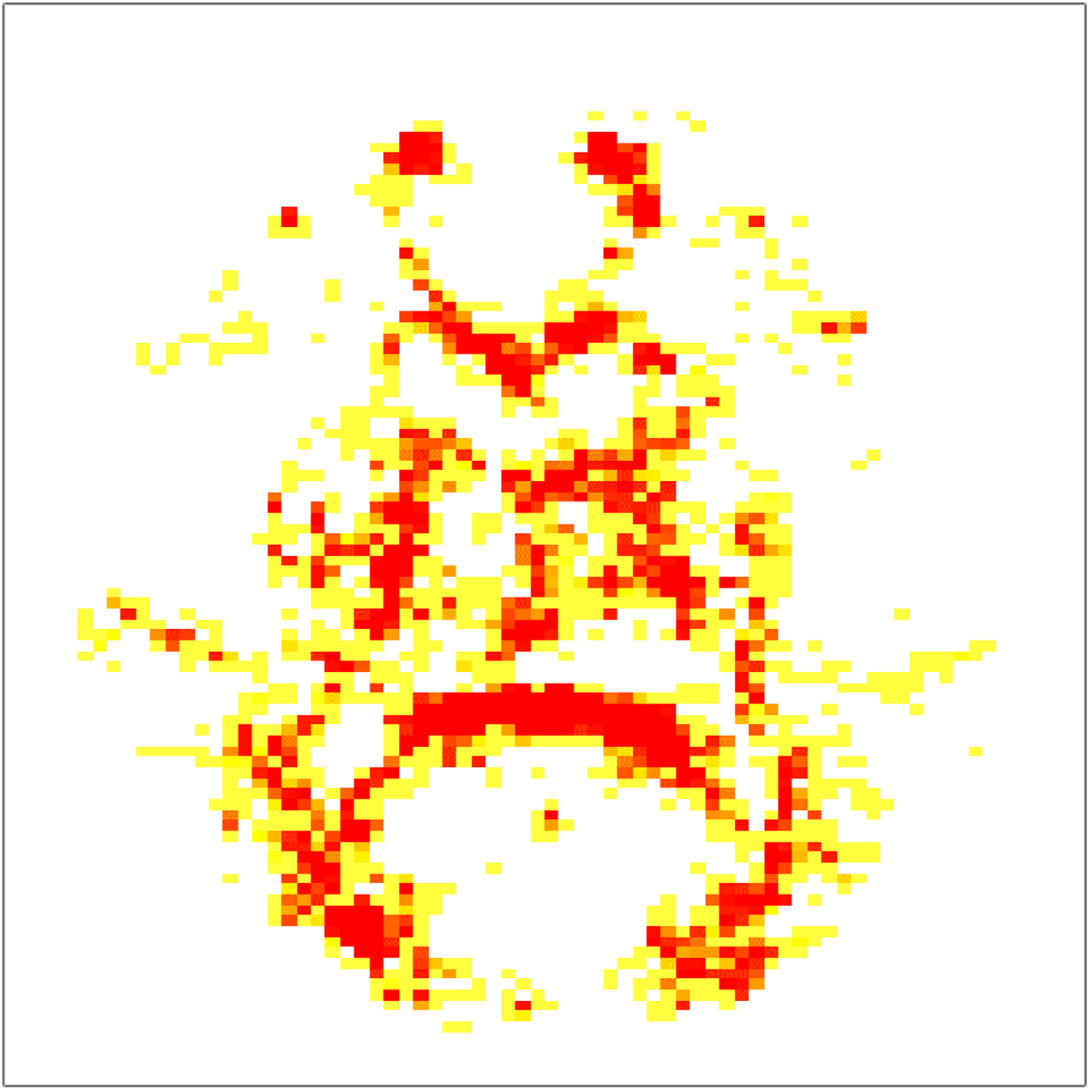
Visualization of adjusted combined *p* value map showing areas of significant injury among all 28 athletes. The yellow/red highlighted regions of the brain are voxels shown to be significantly changed in the athlete from pre-season to post-season (adjusted *p* value < 0.05). Red voxels represent more significant (adjusted *p* value < 0.005) changes than the yellow ones.

When SPREAD results from pre-post comparisons for all 28 athletes were combined into a single *p* value map, we observed the significant changes to be scattered diffusely across the entire white matter region of the brain (Fig. 3).

When viewing all 28-individual *p* value maps, we saw that the SPREAD detected region of WM injury after RHI was highly heterogeneous among the athletes, and we did not identify a contiguous region with significant WM change that is common for all 28 subjects. Illustrative single subject raw *p* value maps can be seen in Fig. 2. All 28 raw *p* value maps can be seen in Supplementary Fig. 2.

Further investigations showed that most of those detected voxels were associated with decreased FA values. For example, using *p* < 0.002 as the *p* value threshold, 106,148 voxels were selected by the SPREAD procedure for all 28 athletes. Among them, 93.3% (99,008 voxels) have lower FA values after the football season. At a more stringent cutoff (*p* < 0.0005), this ratio is even higher: 22,344 voxels were associated with significantly decreased FA values, which made up 95.7% of 23,352 significant voxels.

### Relationship between SPREAD detected WM changes and helmet impact metrics

**Figure 4:**
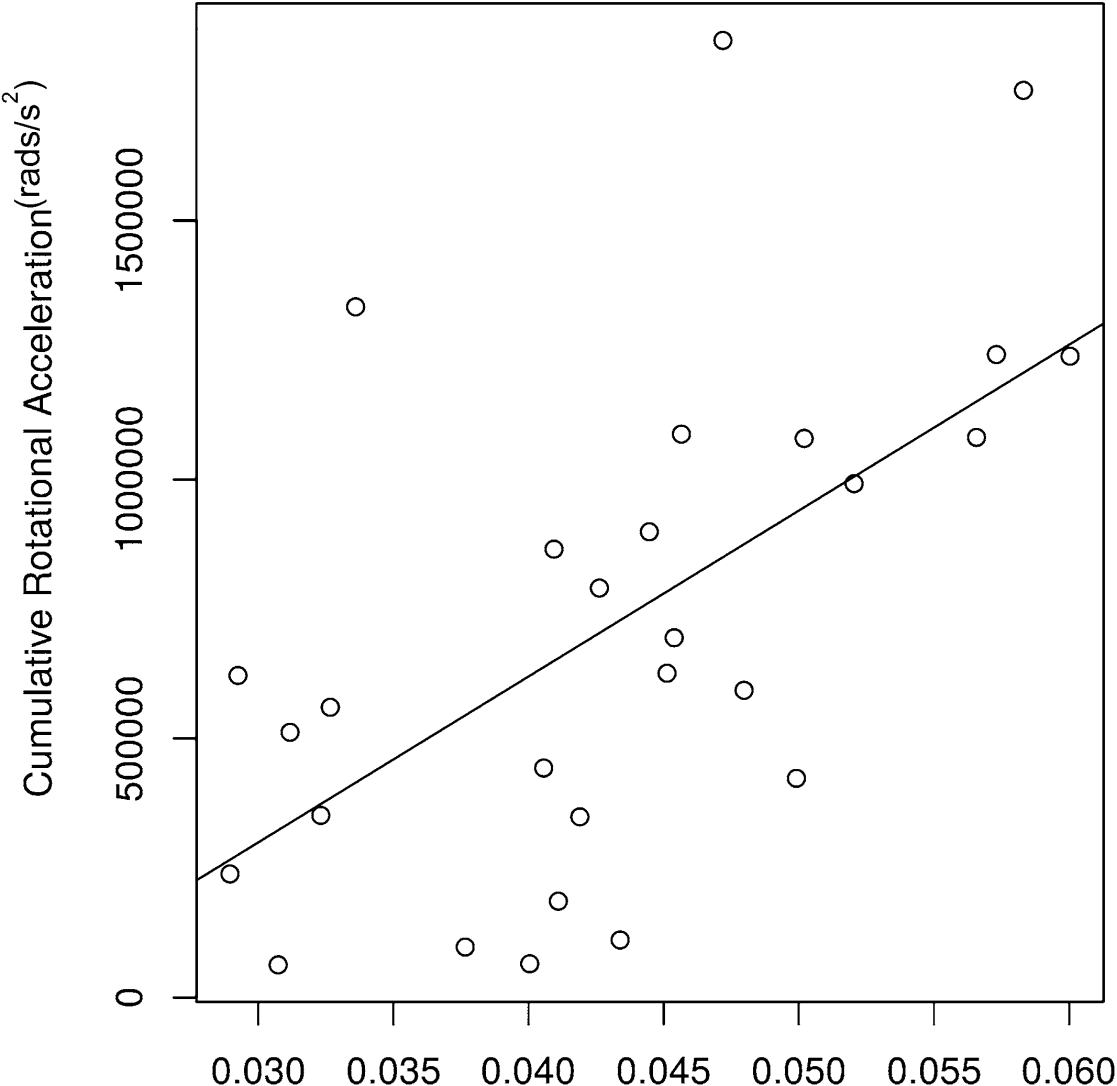
The relationship between the *L*^∞^ summary statistic and CUW rotational acceleration as an illustrative example. This figure depicts the association between the *L*^∞^ summary statistic and the most associative mechanical variable (RA using the CUW metric). The Spearman correlation coefficient was 0.633 with an adjusted *p* value of 0.0049.

The number of head hits over a single season of collegiate football ranged from 37 to 1057 hits with an average of 379 hits per athlete (Supplementary Table 2). We performed correlations between the 30 HIM and helmet impact metric combinations, with the 12 SPREAD derived functional norms and output-bandwidth combinations (Table 3; Supplementary Tables 3 and 4). The most significant correlations with the helmet impact metrics involved using *L*^∞^ norm with a bandwidth of 15. An illustrative example is seen in Fig. 4. In addition, a subset of cumulative time weighted helmet impact metrics is significantly correlated with the *L*^2^ norm with a smaller bandwidth (Supplementary Table 4).

**Table 3:** Helmet impact metric correlations with the ${L}^{\infty }$ norm.

Metric	HIM	Bandwidth 3		Bandwidth 5		Bandwidth 10		Bandwidth 15	
		c.c	Adjusted *p* value	c.c	Adjusted *p* value	c.c	Adjusted *p* value	c.c	Adjusted *p* value
Mean	LA	0.1303	0.8239	0.0640	0.8946	− 0.0224	0.9416	− 0.0126	0.9611
	RA	0.2282	0.8239	− 0.1051	0.8692	− 0.1615	0.6151	0.1724	0.4544
	HIC15	0.1434	0.8239	0.0487	0.9188	− 0.1959	0.4992	− 0.0301	0.9422
	GSI	0.1522	0.8239	0.1155	0.8692	− 0.0794	0.8589	− 0.0099	0.9611
	HITsp	0.0345	0.8916	− 0.1221	0.8692	− 0.0383	0.9070	0.2633	0.2391
Peak	LA	0.0854	0.8239	0.0279	0.9188	− 0.0684	0.8744	0.1527	0.5033
	RA	0.1856	0.8239	0.0328	0.9188	− 0.1067	0.7662	0.1467	0.5052
	HIC15	0.1861	0.8239	0.1582	0.8692	− 0.0487	0.9070	0.2113	0.3490
	GSI	0.1927	0.8239	0.1959	0.8692	− 0.0082	0.9678	0.2452	0.2709
	HITsp	0.0810	0.8239	0.0197	0.9213	− 0.0443	0.9070	0.2742	0.2251
CUW	LA	0.1450	0.8239	0.2124	0.8692	0.5556	**0.0412**	0.6256	**0.0049**
	RA	0.1407	0.8239	0.1845	0.8692	0.5238	**0.0412**	0.6327	**0.0049**
	HIC15	0.1680	0.8239	0.1856	0.8692	0.4592	0.0555	0.5747	**0.0077**
	GSI	0.1872	0.8239	0.2047	0.8692	0.4811	0.0515	0.5692	**0.0077**
	HITsp	0.1319	0.8239	0.1856	0.8692	0.5468	**0.0412**	0.6300	**0.0049**
TBH	LA	0.1987	0.8239	0.1609	0.8692	0.3689	0.1605	0.5665	**0.0077**
	RA	0.1954	0.8239	0.1363	0.8692	0.3372	0.1843	0.5364	**0.0101**
	HIC15	0.2950	0.8239	0.1067	0.8692	0.2167	0.4445	0.5222	**0.0113**
	GSI	0.2879	0.8239	0.1144	0.8692	0.2271	0.4305	0.5052	**0.0144**
	HITsp	0.1658	0.8239	0.1352	0.8692	0.3514	0.1684	0.5868	**0.0077**
TUA	LA	0.0903	0.8239	0.1286	0.8692	0.5161	**0.0412**	0.5599	**0.0077**
	RA	0.0750	0.8239	0.0854	0.8692	0.4669	0.0555	0.5506	**0.0084**
	HIC15	0.0980	0.8239	0.0925	0.8692	0.3623	0.1605	0.4915	**0.0161**
	GSI	0.1253	0.8239	0.1341	0.8692	0.4122	0.1007	0.5238	**0.0113**
	HITsp	0.0722	0.8239	0.1089	0.8692	0.4915	0.0515	0.5594	**0.0077**
TBH + TUA	LA	0.0564	0.8306	− 0.0350	0.9188	0.2485	0.4305	0.4707	**0.0206**
	RA	0.0624	0.8306	− 0.0777	0.8692	0.2326	0.4305	0.4702	**0.0206**
	HIC15	0.1308	0.8239	− 0.0843	0.8692	0.1264	0.7139	0.4308	**0.0364**
	GSI	0.1040	0.8239	− 0.0772	0.8692	0.1253	0.7139	0.4056	**0.0497**
	HITsp	0.0159	0.9368	− 0.0881	0.8692	0.2403	0.4305	0.4975	**0.0155**

“c.c” is the Spearman rank correlation coefficient (ρ). Bold values signify significant correlations (adjusted *p* value < 0.05).

*LA* linear acceleration, *RA* rotational acceleration, *HIC15* head impact criterion 15, *GSI* Gadd Severity Index, *HITsp* helmet impact technology severity profile.

All significant correlations in this association analyses were positive, suggesting that WM structural changes were induced by physical impacts, and the stronger the physical impacts, the greater the maximal injury. The specific metrics that showed significant correlations with *L*^∞^ were the cumulative metrics, all weighted metrics and the CUW metric with Spearman’s ρ values ranging from 0.4056 to 0.6327. The CUW metric had the strongest correlations to *L*^∞^ when compared to all other metrics; however, these differences were small (Table 3).

### Clinical data

We found no significant changes in ImPACT testing scores between pre and post-season testing (Table 4). There was a significant decrease in the BESS test scores with fewer errors seen on average in the post-season evaluation compared to the pre-season evaluation (*p* = 0.003, Table 4). The improvement in the BESS score is most likely a function inter-rater variability as well as a mild practice effect with repeated exposures to the test^38^.

**Table 4:** Mean (SD) ImPACT and BESS performance among contact athletes (n = 28) pre and post-season.

	Pre-season	Post-season	*p* value
Verbal memory score	87 (11)	90 (10)	0.118
Visual memory score	80 (15)	82 (11)	0.268
Visual motor speed	43 (6.3)	44 (6.7)	0.711
Reaction time	0.56 (0.07)	0.56 (0.09)	0.874
BESS total score	20 (9.3)	15 (5.6)	0.003

## Discussion

Our study focused on the ability of SPREAD to identify subject-specific WM changes after one football season, and how SPREAD outputs were associated with multiple single-season summaries of helmet data. It yielded three important findings: (1) SPREAD were able to detect subject-specific FA changes after a single football season; (2) the location of injury is quite heterogeneous and diffuse when looking at the athletes’ injury profiles individually and as a group, respectively; (3) the helmet impact metrics had strong associations with the SPREAD outputs, and more specifically the cumulative metrics appeared to have the strongest associations with the WM changes seen on DTI.

Longitudinal WM changes on DTI have been shown in athletes in multiple studies^15,23^. We detected longitudinal WM changes for the athletes after a single football season, and the identified voxels were overwhelmingly (> 93%) associated with decreased FA values. A decrease in FA is a sign of less unidirectional diffusion and has been reported by others in the context of concussion. The pathological underpinning of decreased FA is controversial but is thought to reflect myelin damage and/or axonal loss^13,39,40^. SPREAD provided a unique opportunity to visualize the injury distribution among the athletes not allowed by previous methods.

Using the SPREAD algorithm and meta-analysis techniques, we were able to create a single common map of injury among all the athlete data. We showed that the athletes as a group had large diffuse areas of injury throughout the white matter of the brain with no distinct, defined region of injury. This diffuse nature is highlighted in other studies that have used region of interest analysis with the individual studies describing different regions with significant change with minimal consistency among the studies^4,17–19^. In comparison, when assessing the athletes individually we found their injury profiles to be quite heterogeneous. This heterogeneous nature of injury within individuals could be a reason why group-level DTI analysis methods have a difficult time showing significant regions of injury as well as a lack of consistent regions of injury among the studies^4,17–19^. The diffuse and heterogeneous nature of this injury suggests group-based level analysis may be a sub-optimal approach for studying RHI induced WM changes, which can be minimized with SPREAD. In addition, this pattern of injury may result from the varying location of impacts among athletes, which minimizes the likelihood of producing similar changes between athletes on DTI. To some extent, these findings explained why some earlier studies failed to establish a clear association between RHI and brain structural changes. The changes are spread out among the entire WM region and different subjects could have quite different changes, so traditional region of interest-based methods could not identify a common region of injury^41,42^. The other advantage SPREAD has provided is the ability to localize injury without requiring a predefined map of brain regions from a specific brain atlas, which allows us to visualize and describe longitudinal WM changes on a more individual level.

The significant correlations between helmet impact metrics and WM changes suggest a dose–response link between RHI and WM changes. The use of helmet data helps create a stronger link because the helmet data is collected prospectively during the time interval WM changes are occurring and being quantified by DTI. Several studies have shown similar patterns when testing the association between helmet impact metrics and WM changes observed on DTI^17,23,24,26,33^. The initial studies done by Bazarian and Davenport showed these associations using unweighted helmet impact metrics^14,26^. In comparison, the strength of the associations improved when the helmet impact data were weighted^17,33^. These studies suggest that other factors affect the biomechanical impact of a hit such as, time between hits, on the WM^17,33^. Looking at our pattern of significant correlations, we found that both CUW and time-weighted metrics had significant correlations even after correcting for multiple comparisons. The cumulative helmet impact metrics being significant suggests these sub-acute WM changes are a result of the compounding effects of a full season of RHI and not associated with a single extreme hit or the average hit experienced by an athlete. More specifically, that time weighted helmet impact metrics were significant suggest that the concept of neuronal vulnerability is important to understanding WM changes in humans, which has been demonstrated in animal models^43,44^. When comparing CUW and time-based metric correlations, the CUW correlations were stronger; however, the differences in Spearman’s $\rho$ were quite small. This finding does not discount the importance of time in understanding the relationship between RHI and WM damage, but suggests our weighting for the time factor could be optimized to the new SPREAD algorithm.

Our examination of clinical data revealed no appreciable declines. The only significant pre to post-season difference was for the BESS scores, which improved over time. This suggests that these clinical measures have a poor sensitivity for the sub-acute WM damage seen on neuroimaging, a finding that has been reported by others^45,46^ In addition, both tests, especially the BESS test, are subject to training effects, making them less useful for detecting subtle neurologic changes that might be associated with sub-acute WM change^38^. The high sensitivity demonstrated by the SPREAD algorithm and its ability to localize injury provides unique opportunities for further research.

The SPREAD algorithm is uniquely suited to identifying the location of injury. Our development of the improved SPREAD (iSPREAD) method provides a more flexible algorithm for analyzing brain scans compared to the SPREAD method^22,47^. iSPREAD uses nonlinear partial differential equation (PDE) modeling techniques to smooth the DTI images, which has better sensitivity and accuracy than SPREAD for detecting changes in regions with irregular shapes. It also has the capability to fit more sophisticated longitudinal models that involve scans with nonlinear temporal trends collected at many time points. Those advantages do come with much higher computational cost and several more tuning parameters in the PDE solver. In the future, we are committed to simplifying the iSPREAD algorithm and providing clinical researchers several practical combinations of tuning parameters via thorough simulation studies. The revised SPREAD method was implemented as an R package, which is freely available at https://github.com/ygu427/iSPREADR.

Future efforts to demonstrate the ability or our iSPREAD method to detect neuroimaging changes might focus on using more sophisticated methods of weighting helmet impact data, such as using machine learning. Another extension would be to utilize the helmet impact location data to help better predict and describe the region of change seen on DTI. Clinically, the ability to measure helmet data could allow the development of threshold values identify athletes who need to temporarily refrain from contact before they develop potentially irreversible or long-term WM changes. This could help lead to improved helmet design as well as protocols to minimize the harmful effects of RHI.

This study is not without limitations. Three of our athletes in our analysis sustained a mid-season concussion, which may provide a confounder to our conclusions; however, the association between the WM changes and physical impacts was not a result of any outliers and the analytic methods used (Spearman’s rank correlation test) was robust to outliers in the data. RHI can be viewed as a spectrum of injuries, with one end being very mild sub-concussive contacts and the other end being clinically defined concussions. In this sense, our current analysis having both concussed and non-concussed athletes may reflect the full extent to which playing football impacts the health of players. Another limitation of our study was the relatively small number of subjects recruited (n = 28). The fact that we were able to show significant associations between the WM changes and helmet impact data despite the small sample size, suggested that using computational methods that are designed to make personalized longitudinal analyses such as, SPREAD. An investigator may be able to detect useful signals from a population with high level of between-subject variation. Should the utility of SPREAD be validated in a future prospective study with a larger sample size, it may potentially change the current research and clinical practice of mTBI in a profound way.

From this study, we can derive three conclusions. The first conclusion is that sub-acute WM changes can be detected by the SPREAD method at the subject-level. Second, we conclude that qualitatively this injury is highly diffuse and heterogeneous among individuals so traditional ROI-based methods may be under-powered. Thirdly, we have shown a significant dose–response relationship between the amount of head trauma a player experiences and the WM changes seen on DTI.

## Supplementary information

Supplementary information
