# Supplementary material for: Spatial regression analysis of MR diffusion reveals subject-specific white matter changes associated with repetitive head impacts in contact sports
Source: Sci Rep. 2020 Aug 12;10:13606. doi: 10.1038/s41598-020-70604-y (PMC7423936; doi:10.1038/s41598-020-70604-y)
Supplement: Supplementary file 1 — Supplementary information [file 41598_2020_70604_MOESM1_ESM.pdf]

Title: Spatial regression analysis of MR diffusion reveals subject-specific white matter changes associated with repetitive head impacts in contact sports

Authors and affiliations:
*Patrick D Asselin[1], MD
[1]Boston Children's Hospital, Department of Pediatrics, 300 Longwood Avenue, Boston, MA 02115, USA

*Yu Gu[2], MS,
[2]University of Rochester, Department of Biostatistics and Computational Biology, 265 Crittenden Blvd, CU 420630 Rochester, New York 14642-0630

Kian Merchant-Borna[3], MPH, MBA
[3]University of Rochester School of Medicine and Dentistry, Department of Emergency Medicine, 265 Crittenden Blvd, Box 655C, Rochester, NY 14642, USA

Beau Abar[3], PhD
[3]University of Rochester School of Medicine and Dentistry, Department of Emergency Medicine, 265 Crittenden Blvd, Box 655C, Rochester, NY 14642, USA

David W. Wright[4], MD
[4]Emory University, Department of Emergency Medicine, 49 Jesse Hill Jr. Drive, Atlanta, GA 30303

Xing Qiu[2], PhD
[2]University of Rochester, Department of Biostatistics and Computational Biology, 265 Crittenden Blvd, CU 420630 Rochester, New York 14642-0630

Jeff J Bazarian[3], MD, MPH
[3]University of Rochester School of Medicine and Dentistry, Department of Emergency Medicine, 265 Crittenden Blvd, Box 655C, Rochester, NY 14642, USA

Supplementary Methods

Details of SPREAD Model used in the current study
In this study, we adapted the original SPREAD pipeline (technical details can be found in [1]) for subject-specific analysis. The statistical model for this approach is represented as follows

$$\mathbb{D}_{ti} = \Phi(z_i) + \beta(z_i) \cdot I\{t = post\} + \epsilon_{ti} \quad \text{for} \quad i = 1, \dots, I \text{ and } t = pre, post. \tag{1}$$

Here $\mathbb{D}_{ti}$ is a set of tensor-derived parameters (e.g., the FA map used in this study) for a subject measured at the pre- or post-scan. z_i represents the 3-dimensional spatial coordinate vector for the ith voxel, $i = 1,2, \dots, I$. The pre-season image (i.e., $\mathbb{D}_{pre,i}$), is modeled as a continuous spatial function $\Phi(z_i)$ superimposed on unknown measurement errors ϵ_{ti} with joint-distribution function $F_\epsilon(\cdot)$. This joint-distribution function does not need to be normal and we only assume it to be invariant under temporal permutation. $I\{t = post\}$ is the indicator function of the post-scan, and the difference between pre- and post-season images, $\beta(z_i)$, is also modeled as a continuous spatial function.

Spatial Regression:
The fitted FA maps were defined as

$$FA_{ti}^{(k)} = \frac{\sum_{j=1}^{I} K_h(z_i - z_j) FA_{ti}}{\sum_{j=1}^{I} K_h(z_i - z_j)}. \tag{2}$$

where z_i, z_j are 3-dimensional coordinate vectors associated with the ith and j^{th} voxel, $K_h(\cdot)$ is a kernel function and h is a tuning parameter which controls its bandwidth. In this study, $K_h(\cdot)$ is the standard Gaussian Kernel function and h is the full width at half maximum (FWHM). The relationship between FWHM and the standard deviation of the Gaussian distribution is $h = 2\sqrt{2ln2}\sigma \approx 2.35\sigma$ voxels. The temporal differences were summarized by the statistic defined as $\Delta FA_i^{(k)} = |FA_{post,i}^{(k)} - FA_{pre,i}^{(k)}|$.

Hypothesis testing:
The null hypothesis is $H_{i0}: \beta(z_i) = 0$, i.e. no significant difference between pre- and post-scan images at the ith voxel. Under the assumption that $F_\epsilon(\cdot)$ is invariant for temporal permutation, permuting the time label of the observation would not affect the distribution function of FA_{ti} if $\beta(z_i) = 0$. Therefore, under the null hypothesis, the detected abnormal region can be defined as the set of voxels at which H_{i0} is rejected by a permutation-based test. Specifically, we randomly permute the time label (pre and post) of each voxel, and refit the spatial regression model (2). The fitted values and the summary of temporal differences are labeled as $FA_{ti}^{(k)}$ and $\Delta FA_i^{(k)}$, respectively, for $k = 1,2, \dots, K$ permutations. We define the permutation-based p-value for the ith voxel as $p_i = \frac{1}{K}\sum_{k=1}^{K} I(\Delta FA_i^{(k)} \geq \Delta FA_i^{(0)})$. Due to the large number of hypotheses (voxels) to be tested, we must use a suitable multiple testing procedure to control for overall type I error. Two MTPs are used in this study: the Benjamini-Hochberg procedure and the Westfall and Young procedure. The first one controls the false discovery rate and the latter controls for family-wise error rate. Both procedures are consistent under positive quadrant correlation and known to work well with permutation-based inferences.

Functional norms:

In this study, we developed several global statistics based on functional norms to summarize the overall temporal changes in DTI images. Recall that after the spatial regression, FA (or other DTI derived quantities such as MD) maps for the sth individual before and after the football season are represented as real valued spatial functions $FA_{s,pre}^{(0)}(\boldsymbol{z})$ and $FA_{s,post}^{(0)}(\boldsymbol{z})$, respectively. Due to the smoothing nature of the Nadaraya-Watson kernel regression and the fact that they are defined on compact domains (the dimensions of the brain images are finite), both $FA_{s,pre}^{(0)}(\boldsymbol{z})$ and $FA_{s,post}^{(0)}(\boldsymbol{z})$ are smooth and bounded, which implies that they are members of functional spaces $L^p(\Omega, \boldsymbol{B}, \mathrm{d}\lambda)$, where $\Omega \subset \boldsymbol{R}^3$ is the image domain, $\boldsymbol{B}$ is the Borel σ-algebra, and $\mathrm{d}\lambda$ is the Lebesgue measure. The L^p norm is a generalization of Euclidean length to functional spaces, which can be defined as follows

$$\|f\|_p := \left(\int_\Omega |f(\boldsymbol{z})|^p \mathrm{d}\,\lambda\right)^{\frac{1}{p}}, \quad 1 \leq p < \infty.$$

As a generalization, it is customary to define the L^∞ norm as the essential supremum of a spatial function as follows

$$\|f\|_\infty := \inf\{C \geq 0 : |f(\boldsymbol{z})|, \text{for } a.e.\, \boldsymbol{z} \in \boldsymbol{R}^3\}.$$

More details of the L^p spaces and L^p norms can be found in most functional analysis textbooks, such as [2].

Meta-analysis:

We conducted a meta-analysis based on a novel robust p-value combination test based on the Beta-distribution. Our objective was to combine p-value maps of all 28 contact athletes into a single group-level p-value map, and then select common change regions based on this single map.

Arguably, the most classical p-value combination method is Fisher's p-value combination test, which can be described as follows,

$$Q_i := -2\sum_{n=1}^{N} \ln p_{ni}, \qquad Q \sim \chi^2_{2N}, \qquad P_i := 1 - F_{\chi^2_{2N}}(Q_i).$$

Here p_{ni}, $n = 1,2,\ldots N$ represent the p-value of voxel i for the nth subject, $F_{\chi^2_{2N}}(Q_i)$ is the distribution function of Chi-squared distribution χ^2_{2N}, and P_i is the combined p-value which follows a uniform distribution on (0,1) under the null hypothesis. However, Fisher's p-value combination test is not suitable in the context of permutation test due to the granularity of permutation p-values. It's obvious that one assumption for Fisher's p-value combination test is that all p_is must be positive, so that $\ln p_{ni}$ is well defined. For permutation tests, p_is may be exactly zero because we cannot use infinitely many permutations, which breaks this assumption. Furthermore, this test is not robust to occasional outliers in the sample, because very small p_{ni}s have disproportionately large impact on the combined p-value: one extremely small p_{ni} (possibly due to outliers in the data) can lead to the overall significance, even if all other subjects are not significant.

As an alternative, we proposed the following robust p-value combination test based on the median p-value map, denoted by $\text{med}(\boldsymbol{p_i})$, where $\boldsymbol{p_i}$ is the vector of p_{ni}s pooled from all subjects. We use the following summary statistic to capture the overall significance of the data

$$S_i := \begin{cases} \text{med}(\boldsymbol{p_i}), & n \text{ is odd} \\ \text{c(n)}\left(\text{med}(\boldsymbol{p_i}) - \frac{1}{2}\right) + \frac{1}{2}, & n \text{ is even} \end{cases}, \quad c(n) \coloneqq \sqrt{\frac{n+1}{n}}, \quad P_i \coloneqq 1 - F_{\text{Beta}(\frac{n+1}{2},\frac{n+1}{2})}(S_i). \tag{3}$$

When n is an odd number, it's well known that $S_i = \text{med}(\boldsymbol{p}_i)$ follows a Beta-distribution $\text{Beta}(\frac{n+1}{2}, \frac{n+1}{2})$ based on the theory of order statistics, therefore we can compute an overall p-value P_i based on the distribution function of $\text{Beta}(\frac{n+1}{2}, \frac{n+1}{2})$. When n is even, the distribution of $\text{med}(\mathbf{p})$ is more complex but it can be proven that the adjusted median, $S_i = \text{c(n)}\left(\text{med}(\boldsymbol{p_i}) - \frac{1}{2}\right) + \frac{1}{2}$, has the same mathematical expectation as $\text{Beta}(\frac{n+1}{2}, \frac{n+1}{2})$, so using the quantile function of $\text{Beta}(\frac{n+1}{2}, \frac{n+1}{2})$ will lead to a well approximated overall p-value in this case.

The algorithm used in our meta-analysis can be summarized as follows
1. Apply affine registration on the fitted difference maps, which is implemented by R package **RNiftyReg**
2. Record the affine transformation matrices and then apply them to the p-value maps.
3. Calculate sample median p-values from all 28 unadjusted p-value maps.
4. Use adjusted Beta-distribution to compute the combined p-value maps.
5. Apply a suitable multiple testing adjustment, such as Holm-Bonferroni procedure which controls familywise error rate, to get the adjusted combined p-value map. Of note, while the WY procedure also controls for the family-wise error rate, it is a permutation-based MTP which is not directly applicable to p-value combination test.
6. Select significant voxels based on this adjusted combined p-value map, which is presented in Fig. 3.

Supplementary Fig. 1: Examples of SPREAD Parameter Combinations

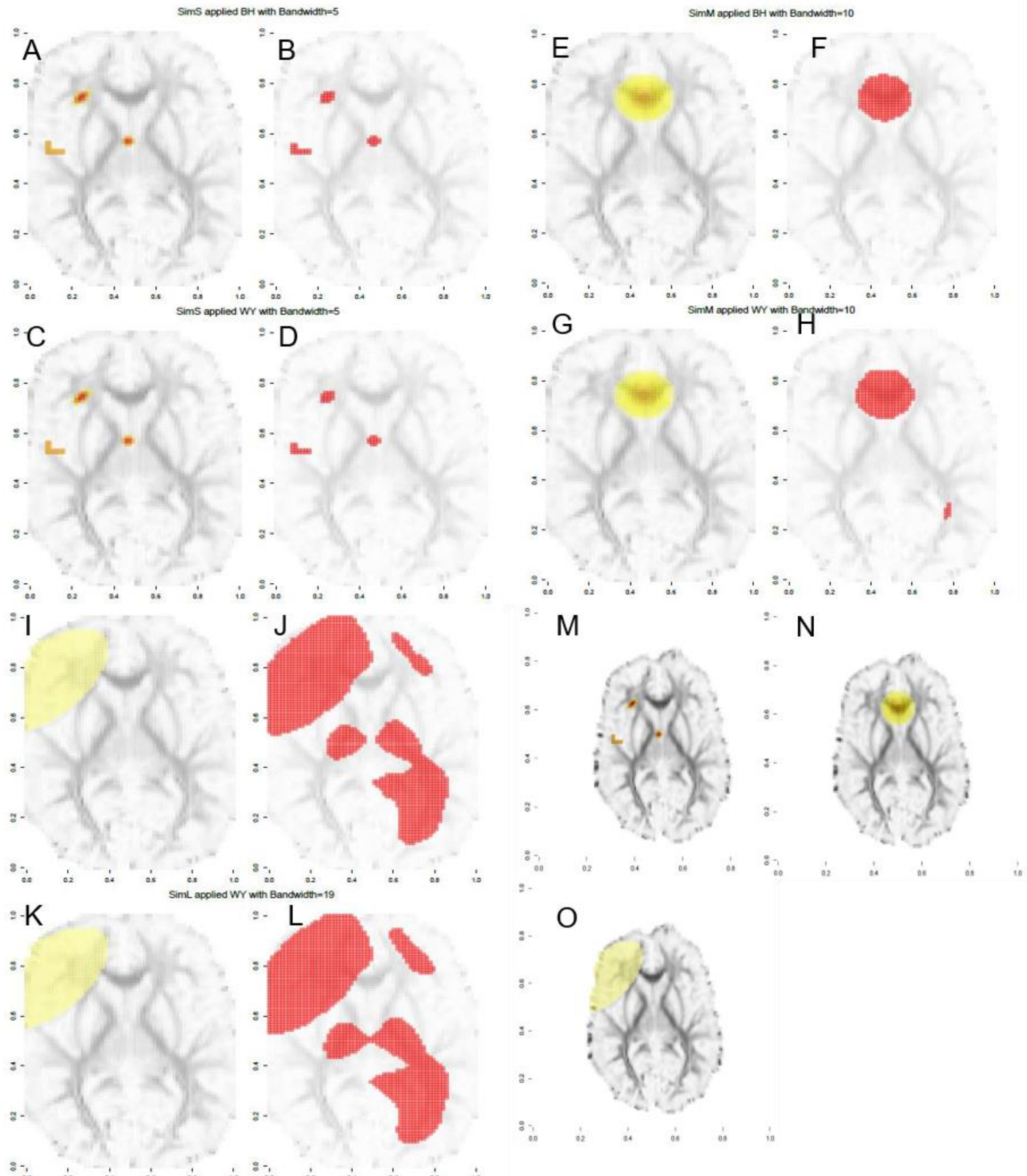

(A) A visualization of the actual small synthesized signal created in the simulation study. (B) Region of significantly changed voxels detected by SPREAD at a smoothing bandwidth of 5 and using the Benjamini-Hochberg procedure. (C) A visualization of the actual small synthesized signal created in the simulation study. (D) Region of significantly changed voxels detected by SPREAD at a smooth bandwidth of 5 and using the Westfall-Young procedure. (E) A visualization of the actual medium synthesized signal created in the simulation study. (F) Region of significantly changed voxels detected by SPREAD at a smoothing bandwidth of 10 and using the Benjamini-Hochberg procedure. (G) A visualization of the actual medium synthesized signal created in the simulation study. (H) Region of significantly changed voxels detected by SPREAD

at a smooth bandwidth of 10 and using the Westfall-Young procedure. (I) A visualization of the actual large synthesized signal created in the simulation study. (J) Region of significantly changed voxels detected by SPREAD at a smoothing bandwidth of 19 and using the Benjamini-Hochberg procedure. (K) A visualization of the actual large synthesized signal created in the simulation study. (L) Region of significantly changed voxels detected by SPREAD at a smooth bandwidth of 19 and using the Westfall-Young procedure. (M) A visualization of the small synthesized signal from the simulation study. (N) A visualization of the medium synthesized signal from the simulation study. (O) A visualization of the large synthesized signal from the simulation study.

Supplementary Fig. 2. All athletes' individual raw p-value map at registered slice 32.

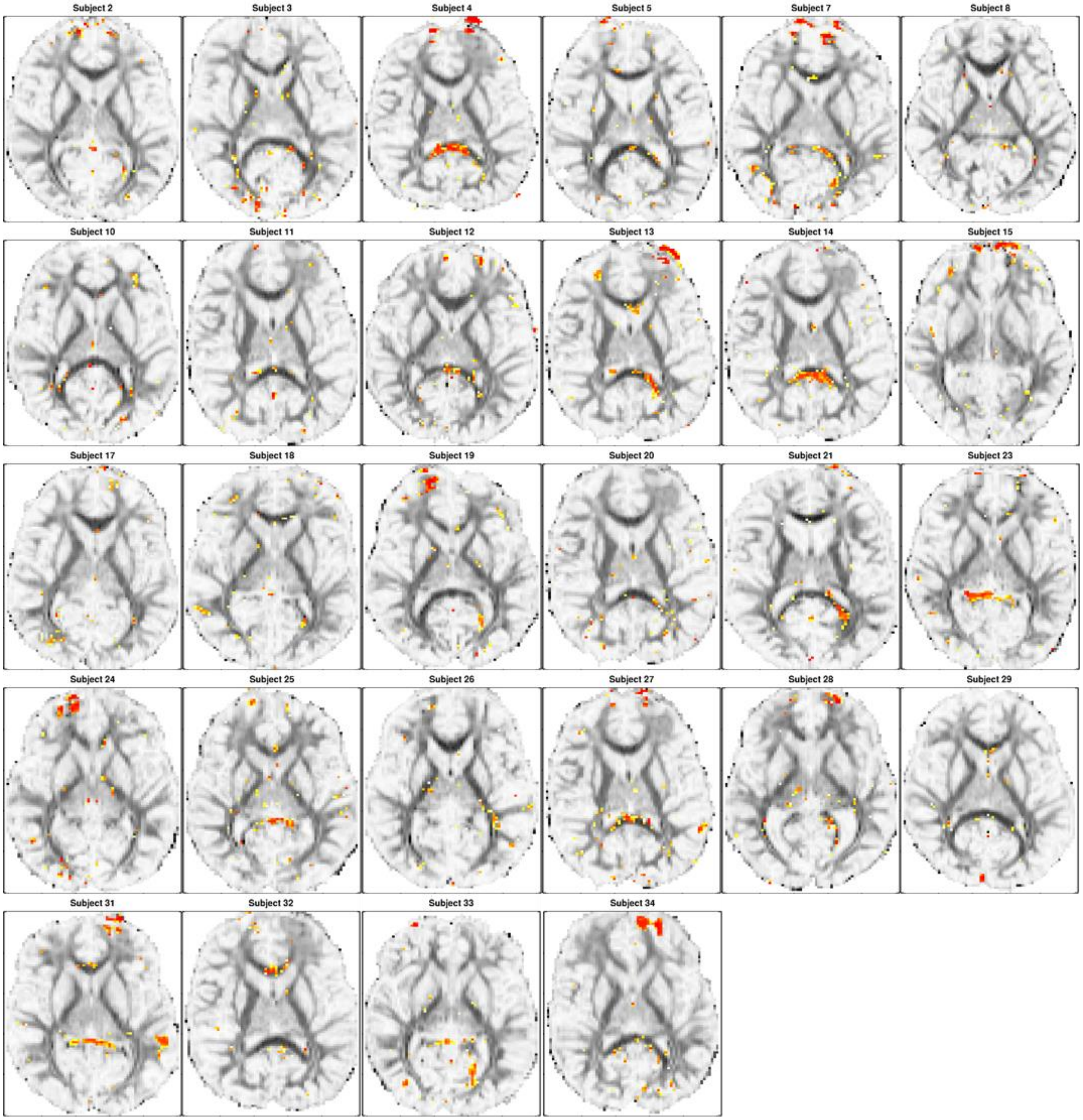

The yellow/red highlighted regions of the brain are voxels with associated raw p-values of <0.002 when comparing the pre-season to post-season scans. The red voxels are associated with a lower raw p-value than the yellow ones.

Supplementary Fig. 3. Significant ($p < 0.002$) temporal changes of FA values (post-season – pre-season) for all athletes at registered slice 32. Blue color is used to represent decreased FA values and red represents increased FA values. The overwhelming majority (93.3%) of significant voxels have decreased FA values after the football season.

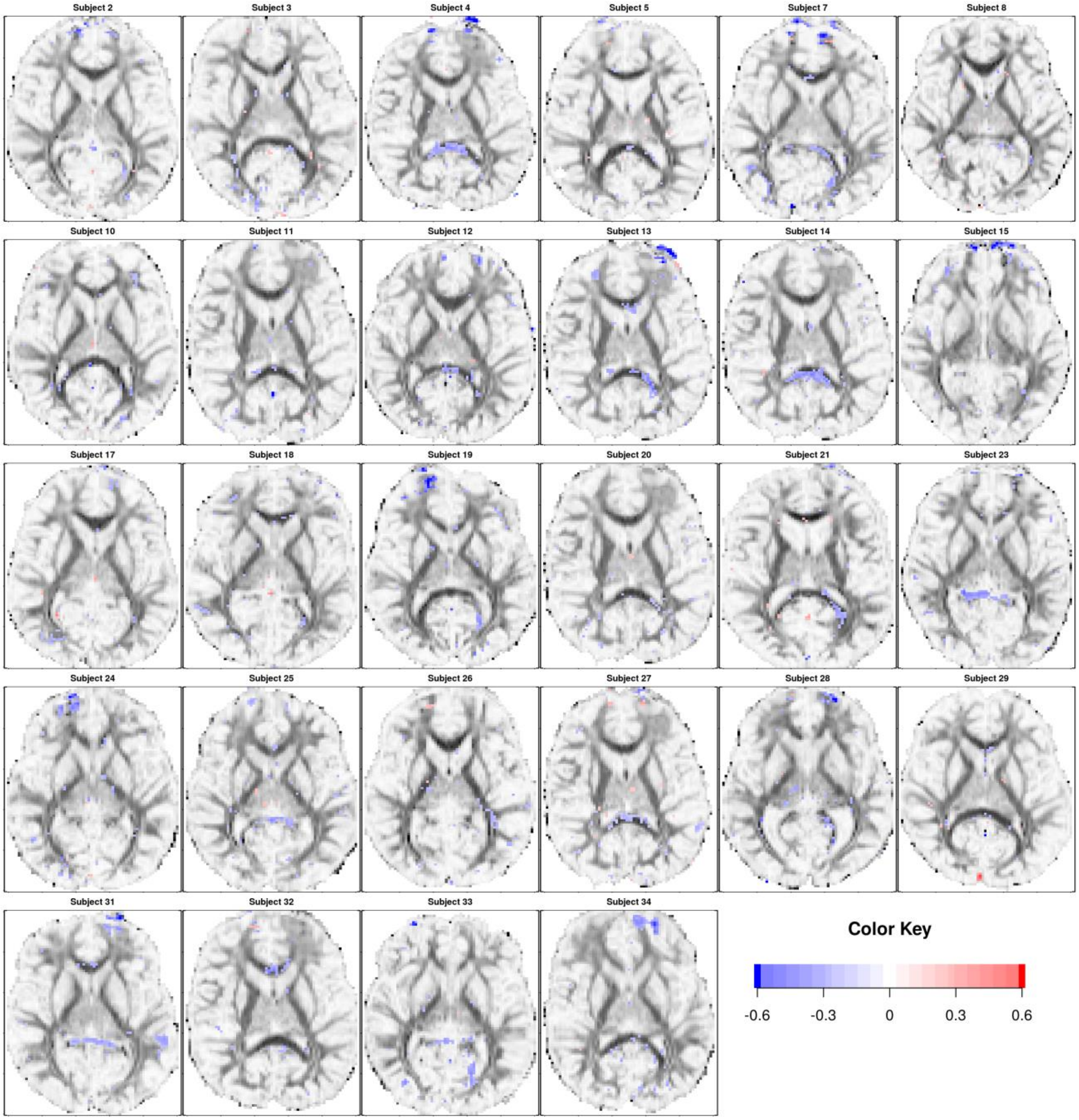

Supplementary Fig. 4: Removal of the ringing artifacts

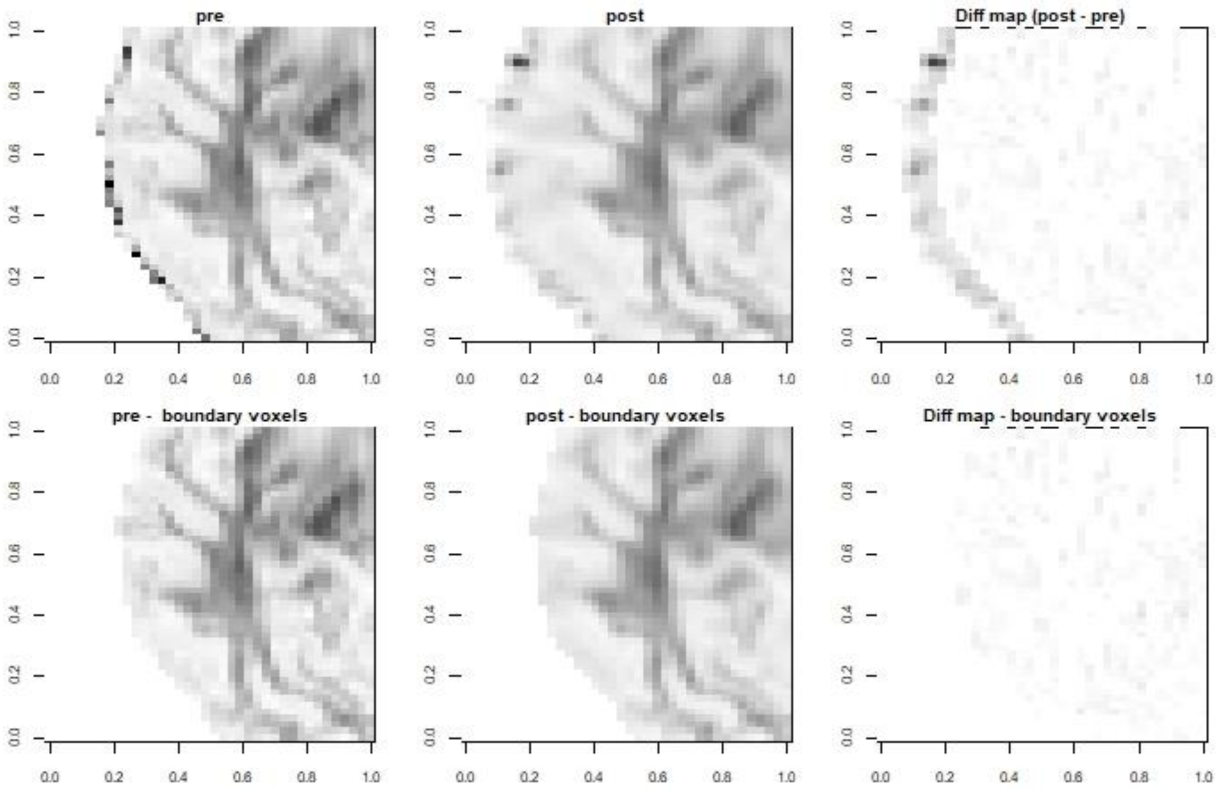

An illustration of the ringing artifacts and our strategy of removing them. First, we detect the foreground and background of the FA map by a pre-specified intensity threshold such as 0.01. Next, we enlarge the background mask by a pre-specified Euclidean distance such as two voxels in all 26 spatial directions. The final foreground mask is defined as the set complement of the enlarged background map. This procedure is implemented as function MountDoom() in our software package.

Supplementary Table 1. Basic demographics for all contact athletes and controls

ID #	Age	Handedness	Race	Position	Mid-season concussion	Comment
2	21.8	Right	Other	Strong Safety	0	
3	19.1	Right	Black	Outside Line Backer	0	
4	18.6	Right	White	Offensive Lineman	0	
5	20.1	Right	White	Outside Line Backer	0	
6	19.1	Right	White			Excluded PIP*
7	19.6	Right	White	Wide Receiver	0	
8	22.0	Left	White	Corner Back	0	
10	18.9	Right	White	Defensive Tackle	0	
11	21.6	Left	Black	Wide Receiver	0	
12	21.1	Right	White	Defensive End	0	
13	21.5	Right	White	Defensive Tackle	0	
14	19.7	Right	White	Defensive End	0	
15	19.9	Right	White	Inside Line Backer	0	
17	21.5	Right	White	Tight End	1	
18	19.4	Right	Black	Free Safety	0	
19	21.3	Left	White	Defensive End	0	
20	21.0	Right	White	Offensive Lineman	0	
21	19.0	Right	Black	Corner Back	0	
23	18.9	Left	White	Outside Line Backer	0	
24	19.0	Right	White	Strong Safety	0	
25	21.6	Right	White	Offensive Lineman	1	
26	21.4	Left	Other	Running Back	0	
27	21.6	Right	White	Strong Safety	0	
28	19.9	Right	White	Outside Line Backer	0	
29	19.7	Right	White	Corner Back	0	
31	18.8	Right	Black	Running Back	1	
32	19.6	Right	White	Tight End	0	
33	19.6	Right	White	Running Back	0	
34	19.7	Left	White	Offensive Lineman	0	

* PIP is poor DTI image processing

Supplementary Table 2: Summary of helmet-based impact measures. LA: linear acceleration; RA: rotational acceleration; HIC15: head impact criterion 15; GSI: Gadd Severity Index; HITsp: helmet impact technology severity profile.

Exposure	Mean	Min	Max
Total Hits	379	37	1057
Mean			
**LA**	30	24	36
**RA**	1931	1504	2288
**HIC 15**	20	10	30
**GSI**	30	15	46
**HITsp**	18	15	20
Peak			
**LA**	134	67	227
**RA**	9213	5030	15217
**HIC 15**	416	63	1096
**GSI**	597	100	1411
**HITsp**	97	36	229
CUW			
**LA**	11562	885	31885
**RA**	719341	63021	1847651
**HIC 15**	7221	371	19600
**GSI**	11022	581	30732
**HITsp**	6563	572	17273
TBH			
**LA**	718029406	20591682	2029992077
**RA**	45091564064	1361704123	115032650410
**HIC 15**	601069851	5117060	2326720102
**GSI**	890000716	6600590	3511226283
**HITsp**	393522625	16414424	1076179853
TUA			
**LA**	283	21	812
**RA**	18075	1476	47729
**HIC 15**	172	10	519
**GSI**	261	15	813
**HITsp**	162	14	443
TBH+TUA			
**LA**	20611549	332134	70455643
**RA**	1384018628	21405493	5860030308
**HIC 15**	15664952	84202	62212765
**GSI**	22926740	112721	95355128
**HITsp**	10931072	254411	30023615

Supplementary Table 3. Correlations between helmet impact metrics and L^1 bandwidth combinations. LA: linear acceleration; RA: rotational acceleration; HIC15: head impact criterion

15; GSI: Gadd Severity Index; HITsp: helmet impact technology severity profile. “c.c” is the Spearman rank correlation coefficient (ρ).

		Bandwidth 3		Bandwidth 5		Bandwidth 10		Bandwidth 15	
Metric	HIM	c.c.	adjusted p-value	c.c.	adjusted p-value	c.c.	adjusted p-value	c.c.	adjusted p-value
Mean	LA	0.0454	1.0000	0.1138	0.5859	0.1232	0.5308	0.1368	0.4858
Mean	RA	0.0668	1.0000	0.2414	0.3898	0.3525	0.1573	0.3870	0.1942
Mean	HIC15	0.0213	1.0000	0.1478	0.5415	0.1888	0.3715	0.1779	0.4039
Mean	GSI	0.0564	1.0000	0.1642	0.5026	0.2080	0.3586	0.2058	0.3651
Mean	HITsp	0.0312	1.0000	0.2217	0.3898	0.3695	0.1573	0.4193	0.1942
Peak	LA	0.0274	1.0000	0.1128	0.5859	0.1609	0.4259	0.1593	0.4309
Peak	RA	0.0000	1.0000	0.0925	0.6385	0.1686	0.4173	0.1675	0.4207
Peak	HIC15	0.0044	1.0000	0.1237	0.5859	0.2014	0.3632	0.1932	0.3877
Peak	GSI	0.0148	1.0000	0.1352	0.5667	0.1888	0.3715	0.1866	0.3924
Peak	HITsp	0.0881	0.9351	0.2222	0.3898	0.2813	0.2178	0.2846	0.2365
CUW	LA	0.3711	0.3077	0.3612	0.2441	0.3443	0.1573	0.3262	0.1942
CUW	RA	0.3519	0.3077	0.3503	0.2441	0.3574	0.1573	0.3481	0.1942
CUW	HIC15	0.3897	0.3077	0.4034	0.2441	0.3968	0.1573	0.3908	0.1942
CUW	GSI	0.4149	0.3077	0.4149	0.2441	0.3935	0.1573	0.3875	0.1942
CUW	HITsp	0.3558	0.3077	0.3503	0.2441	0.3476	0.1573	0.3355	0.1942
TBH	LA	0.2742	0.3715	0.3383	0.2441	0.3695	0.1573	0.3421	0.1942
TBH	RA	0.2315	0.4696	0.2989	0.2520	0.3519	0.1573	0.3361	0.1942
TBH	HIC15	0.2895	0.3680	0.3870	0.2441	0.4023	0.1573	0.3530	0.1942
TBH	GSI	0.2720	0.3715	0.3629	0.2441	0.3815	0.1573	0.3415	0.1942
TBH	HITsp	0.2540	0.4104	0.3333	0.2441	0.3727	0.1573	0.3563	0.1942
TUA	LA	0.3662	0.3077	0.3273	0.2441	0.3158	0.2037	0.2917	0.2365
TUA	RA	0.3290	0.3077	0.3010	0.2520	0.3010	0.2052	0.2781	0.2388
TUA	HIC15	0.2901	0.3680	0.3082	0.2520	0.3443	0.1573	0.3279	0.1942
TUA	GSI	0.3246	0.3077	0.3333	0.2441	0.3563	0.1573	0.3426	0.1942
TUA	HITsp	0.3262	0.3077	0.2961	0.2520	0.2983	0.2052	0.2846	0.2365
TBH+TUA	LA	0.1554	0.7806	0.2167	0.3898	0.2775	0.2178	0.2682	0.2388
TBH+TUA	RA	0.1494	0.7806	0.2206	0.3898	0.3071	0.2052	0.3032	0.2337
TBH+TUA	HIC15	0.1275	0.8151	0.2042	0.4036	0.2677	0.2292	0.2534	0.2625
TBH+TUA	GSI	0.1111	0.8581	0.1856	0.4473	0.2507	0.2576	0.2348	0.2975
TBH+TUA	HITsp	0.1423	0.7806	0.2140	0.3898	0.2802	0.2178	0.2709	0.2388

Supplementary Table 4. Correlations between helmet impact metric with L^2 bandwidth combinations. LA: linear acceleration; RA: rotational acceleration; HIC15: head impact criterion 15; GSI: Gadd Severity Index; HITsp: helmet impact technology severity profile. “c.c” is the Spearman rank correlation coefficient (ρ). Bold values signify significant correlations (adjusted p-value <0.05).

		**Bandwidth 3**		**Bandwidth 5**		**Bandwidth 10**		**Bandwidth 15**	
Metric	HIM	c.c.	adjusted p-value	c.c.	adjusted p-value	c.c.	adjusted p-value	c.c.	adjusted p-value
Mean	LA	0.1500	0.4763	0.1418	0.4863	0.1576	0.4214	0.1615	0.4101
	RA	0.1522	0.4763	0.2490	0.2506	0.3596	0.0830	0.3979	0.1340
	HIC15	0.1215	0.5549	0.1275	0.5162	0.2085	0.2954	0.1943	0.3314
	GSI	0.1680	0.4512	0.1719	0.4074	0.2271	0.2639	0.2239	0.2869
	HITsp	0.1002	0.6108	0.2288	0.2775	0.3700	0.0830	0.4116	0.1340
Peak	LA	0.2698	0.2146	0.2496	0.2506	0.2518	0.2255	0.2206	0.2869
	RA	0.2080	0.3442	0.1954	0.3528	0.2261	0.2639	0.2124	0.2964
	HIC15	0.2365	0.2810	0.2370	0.2684	0.2852	0.1694	0.2359	0.2711
	GSI	0.2857	0.1914	0.2693	0.2255	0.2917	0.1649	0.2469	0.2557
	HITsp	0.3076	0.1611	0.3229	0.1428	0.3673	0.0830	0.3465	0.1340
CUW	LA	0.4997	0.0546	0.4915	**0.0464**	0.4143	0.0732	0.3558	0.1340
	RA	0.4959	0.0546	0.4981	**0.0464**	0.4330	0.0732	0.3755	0.1340
	HIC15	0.5583	**0.0356**	0.5523	**0.0404**	0.4817	0.0732	0.4275	0.1340
	GSI	0.5676	**0.0356**	0.5545	**0.0404**	0.4718	0.0732	0.4171	0.1340
	HITsp	0.4882	0.0546	0.4871	**0.0464**	0.4215	0.0732	0.3645	0.1340
TBH	LA	0.4401	0.0613	0.4669	**0.0464**	0.4532	0.0732	0.3870	0.1340
	RA	0.4182	0.0693	0.4521	**0.0497**	0.4412	0.0732	0.3859	0.1340
	HIC15	0.4576	0.0613	0.4729	**0.0464**	0.4691	0.0732	0.3941	0.1340
	GSI	0.4494	0.0613	0.4631	**0.0464**	0.4587	0.0732	0.3870	0.1340
	HITsp	0.4379	0.0613	0.4729	**0.0464**	0.4641	0.0732	0.4094	0.1340
TUA	LA	0.4324	0.0613	0.4149	0.0727	0.3706	0.0830	0.3016	0.1698
	RA	0.4067	0.0700	0.3941	0.0777	0.3645	0.0830	0.2972	0.1698
	HIC15	0.4012	0.0705	0.4056	0.0765	0.4198	0.0732	0.3591	0.1340
	GSI	0.4346	0.0613	0.4346	0.0593	0.4286	0.0732	0.3706	0.1340
	HITsp	0.4116	0.0700	0.3985	0.0777	0.3656	0.0830	0.3060	0.1698
TBH+TUA	LA	0.3262	0.1611	0.3388	0.1331	0.3695	0.0830	0.3213	0.1607
	RA	0.3251	0.1611	0.3519	0.1255	0.3897	0.0830	0.3498	0.1340
	HIC15	0.3131	0.1611	0.3218	0.1428	0.3602	0.0830	0.3060	0.1698
	GSI	0.3065	0.1611	0.3114	0.1527	0.3470	0.0926	0.2868	0.1810
	HITsp	0.3153	0.1611	0.3372	0.1331	0.3684	0.0830	0.3207	0.1607

References

1. Zhu, T., et al., *Spatial regression analysis of diffusion tensor imaging (SPREAD) for longitudinal progression of neurodegenerative disease in individual subjects.* Magnetic resonance imaging, 2013. **31**(10): p. 1657-1667.
2. Conway, J.B., *A course in functional analysis*. 1990: Springer.
